# Active Surveillance of Small Renal Masses in a Large Danish Cohort: Assessing Proficiency in Patient Selection

**DOI:** 10.15586/jkcvhl.v11i1.318

**Published:** 2024-03-28

**Authors:** Rasmine Bak, Jørgen Bjerregaard Jensen, Tau Pelant, Rikke Nørresø Haase, Tommy Kjærgaard Nielsen

**Affiliations:** 1 Department of Urology, Aarhus University Hospital, Aarhus, Denmark;; 2 Department of Clinical Medicine, Aarhus University, Aarhus, Denmark;; 3 Department of Urology, Regional Hospital Gødstrup, Gødstrup, Denmark;; 4 Department of Urology, Aalborg University Hospital, Aalborg, Denmark

**Keywords:** active surveillance, delayed intervention, kidney cancer, small renal masses

## Abstract

Small renal masses (SRMs) are often benign or early-stage cancers with low metastatic potential. The risk of overtreating SRMs is a particular concern in elderly or comorbid patients, for whom the risks associated with active surveillance (AS) are lower than the risks of surgical management. The aim is to systematically analyse a large cohort of AS patients to provide valuable insights into patient selection and outcomes concerning delayed intervention (DI) and AS termination. We retrospectively analysed data from 563 AS patients across three institutions from 2012 to 2023. Patients were classified into three groups: those currently in AS (n=283), those who underwent DI (n=75), and those who terminated AS (n=205). DI patients were younger, and had larger initial tumour size and higher growth rates (GRs) than AS patients. A significant number of patients terminated their AS, mainly due to comorbidities and death from non-kidney cancer causes, suggesting unsuitability for initial AS enrolment. AS appears to be a safe initial management strategy for SRMs, with an overall low GR and only one patient developing metastasis. The patient selection for AS appears inconsistent, highlighting the need for improved criteria to identify AS candidates, especially considering comorbidities and the possibility of subsequent active treatment in the event of progression.

## Introduction

The incidence of small renal masses (SRMs) has increased over the last three decades due to a widespread increase in the use of computed tomography (CT) (1). This has led to stage migration and more frequent incidental detection of SRMs in asymptomatic and elderly patients (2). SRMs are defined as tumours <4 cm with suspicion of being renal cell carcinoma (3). SRMs represent a heterogeneous group of tumours, varying from benign lesions and early-stage tumours to aggressive lesions with metastatic potential. On average, 20% of SRMs are benign, 60% are indolent cancers, and up to 20% are potentially aggressive cancers (4).

The risk of overtreating SRMs is especially concerning in elderly and comorbid patients, for whom the risk associated with active surveillance (AS) is often lower than the risk of surgical management (5). AS is not synonymous with observation or watchful waiting, but is defined as initial management, including an individualised follow-up strategy, with serial imaging to monitor tumour size. In case of progression, defined as tumour size >4 cm, growth rate (GR) >5 mm/yr, symptoms, metastasis or elective crossover, which refers to a change in patients’ preference or improvement in health, a course of delayed treatment is indicated (3). AS is widely accepted as a safe option for patients with SRMs <2 cm or for larger tumours in patients with advanced age, comorbidities or a strong personal preference (3).

No standardised protocols have been established for patient selection or assessing the risk of progression during AS. Thus, the decision of AS relies on a complex range of factors, including patient age and comorbidities, tumour characteristics and patient preference regarding treatment options (6). The existing literature and knowledge about SRMs and AS are largely derived from retrospective studies. To date, only a few studies have evaluated AS protocols prospectively (7–15), and a randomised trial is yet to be conducted. The present study aimed to systematically analyse a large cohort of AS patients to provide valuable insights into patient selection and outcomes concerning delayed intervention (DI) and AS termination. Despite its retrospective nature, this study provides essential information because it is the first study to examine patients with a terminated AS protocol.

## Materials and Methods

### Study population

Patients in an AS programme at Aarhus University Hospital from 2012 to 2023 and at Regional Hospital Gødstrup and Aalborg University Hospital from 2018 to 2023 were included in this study. The variations in timeframe among the three centres were due to differences in the availability of the electronic medical journal system and ethical approval from their respective institutions. Relevant patients were identified through a combination of the Electronic Medical Journal System and Business Intelligence System by referrals, diagnosis and treatment codes. All patients with at least one follow-up during the specified period were included. Patients with hereditary syndromes or diagnosis of renal angiomyolipoma, oncocytoma or Bosniak 2F or lower-grade cysts were not included in the analysis.

### Clinical data

The follow-up time was defined as the duration from the AS decision to the last follow-up imaging, the decision of DI or the termination of AS. The overall GR was calculated as the size difference between the first and last scans divided by the time interval between the two scans. The GR was expressed as the rate of change in millimetres per year. Comorbidities were assessed using the age-adjusted Charlson Comorbidity Index (CCI) Score. In cases in which the patient had more indications for AS or termination of AS, the primary indication was considered.

### Statistics

Categorical variables were compared using Pearson’s chi-squared, whereas continuous variables were assessed using one-way ANOVA. Before conducting the ANOVA, Bartlett’s test was performed to check for equal variances. In terms of unequal variances, the data were either log-transformed or assessed using the Mann–Whitney test. Kruskal–Wallis test was used to determine significant differences between medians. P<0.05 was considered significant. All statistical analyses were conducted using RStudio version 2022.07.2+576.

## Results

A total of 563 patients were retrospectively identified and categorised into three groups: those currently enrolled in an AS programme (n=283), those who underwent DI (n=75) and those who terminated AS (n=205).

### Demographics

The final analysis included 563 patients with a total of 616 tumours (mean age at diagnosis 73 years). The majority of the patients were male (62%). The primary indications for AS were comorbidity (37%) and a tumour too small for further initial diagnosis (26%). Other indications for AS included an inconclusive biopsy (11%), low malignancy type (8%), patient preference (7%), treatment of non-kidney cancer at the time of diagnosis (6%) and advanced age (5%). Patients who terminated AS were older at diagnosis and had a higher comorbidity burden than the AS and DI groups (p<0.001). Patients currently enrolled in an AS programme presented with a smaller median initial tumour size than the other two groups (13 mm vs. 18 mm for DI and 16 for terminated AS; p<0.001), but with a similar tumour size range (p=0.368). The median overall follow-up was 14 months, with no significant differences among the three groups (p=0.368). The minimum overall follow-up was 1.9 months, with no significant differences among the three groups (p=0.368). The overall median GR was 0 mm/yr, with a significantly higher median GR in the DI group (3 mm/yr) compared to both the AS and terminated AS group (0 mm/yr; p<0.001). No significant differences were observed in the range of GR among the three groups (p=0.368). There were no differences regarding smoking status, prior surgery status, single kidney status or tumour location among the three groups. An initial biopsy was performed in 33% of patients, with a diagnostic rate of 64%. Significant differences were observed among the three groups in terms of Fuhrman grade (p=0.041) and histology (p=0.028). However, the diagnostic rate was uneven in the three groups. A total of 9.3% were diagnosed with Bosniak cyst grade III or IV, with a higher prevalence observed in the DI group (13.5% vs. 10% for the AS group and 6.8% for terminated AS; p=0.027). The majority of patients (80%) were discussed at a Multidisciplinary Team Conference (MDT) before entering AS. CT was initially used in 96% of cases, with 94% of the SRMs being incidental findings. No major discrepancies were noted by the different centres. Patient demographics and tumour characteristics are presented in Table 1.

**Table 1:** Patient and tumour characteristics for patients currently enrolled in an active surveillance (AS) programme, patients who underwent delayed intervention (DI) and patients who terminated AS.

	Overall (n = 563)	AS (n = 283)	DI (n = 75)	Terminated AS (n = 205)	p-value
Mean age (SD)	73 (9.4)	72 (8.7)	69 (9.8)	76 (9.3)	<0.001
No. male, n (%)	349 (62.2)	178 (63.1)	51 (68.0)	120 (58.8)	0.339
Mean BMI (SD)	27 (5.6)	27 (5.6)	28 (5.3)	26 (5.7)	0.0307
Mean initial creatinine µmol/L (SD)	98.8 (75.8)	96.3 (62.2)	94.3 (90.7)	103.8 (86.0)	0.353
Mean a-CCI score (SD)	5.7 (2.8)	5.1 (2.6)	5.4 (2.5)	6.7 (2.8)	<0.001
a-CCI score, n (%)	<0.001				
0–5	296 (52.6)	179 (63.3)	39 (52.0)	78 (38.0)	
6–10	231 (41.0)	93 (32.9)	33 (44.0)	105 (51.2)	
≥10	36 (6.4)	11 (3.9)	3 (4.0)	22 (10.7)	
Smoking status, n (%)	0.343				
Current smoker	120 (23.3)	59 (23.5)	20 (28.2)	41 (21.1)	
Never smoker	189 (36.6)	83 (33.1)	27 (38.0)	79 (40.7)	
Former smoker	207 (40.1)	109 (43.4)	24 (33.8)	74 (38.1)	
Prior surgery, n (%)					
Kidney	33 (4.2)	16 (3.3)	6 (6.8)	11 (4.4)	0.397
Abdominal	190 (32.7)	98 (33.7)	23 (29.7)	69 (32.3)	0.805
Single kidney, n (%)	22 (2.9)	9 (2.1)	2 (1.4)	11 (4.4)	0.232
Laterality, n (%)	0.049				
Right	255 (45.3)	137 (48.4)	41 (54.7)	77 (37.6)	
Left	266 (47.2)	124 (43.8)	31 (41.3)	111 (54.1)	
Multiple	42 (7.5)	22 (7.8)	3 (4.0)	17 (8.3)	
Median initial tumour size, mm (range)	15 (4-90)	13 (4-75)	18 (7-75)	16 (5-90)	<0.001
Initial tumour size, n (%)	0.001				
< 20 mm	423 (68.7)	234 (75.5)	46 (59.0)	143 (62.7)	
20–30 mm	105 (17.0)	40 (12.9)	22 (28.2)	43 (18.9)	
≥ 30 mm	88 (14.3)	36 (11.6)	10 (12.8)	42 (18.4)	
Median follow-up time, month (range)	14.4 (1.9-118)	18.2 (1.9-104)	11.9 (2.1-77)	13.2 (3.9-118)	0.368
Median GR, mm/yr (range)	0 (-37-68)	0 (-13-68)	3 (-4-68)	0 (-37-34)	<0.001
Initial biopsy, n (%)	185 (32.9)	90 (31.8)	35 (47.3)	60 (29.3)	0.016
T1b, n (%)	13 (7.0)	5 (5.6)	3 (8.6)	5 (8.3)	0.315
Fuhrman, n (%)					0.041
Grade 1–2	99 (53.5)	42 (46.7)	25 (71.4)	32 (53.3)	
Grade 3–4	10 (5.4)	3 (3.3)	3 (8.6)	4 (6.7)	
Non-diagnostic biopsy	76 (41.1)	45 (50.0)	7 (20.0)	24 (40.0)	
Histology, n (%)					0.028
Clear Cell	50 (27.0)	17 (18.9)	17 (48.6)	16 (26.7)	
Chromophobe	9 (4.9)	5 (5.6)	0 (0.0)	4 (6.7)	
Papillary	42 (22.7)	19 (21.1)	9 (25.7)	14 (23.3)	
Other histology	17 (9.2)	12 (13.3)	2 (5.7)	3 (5.0)	
Non-diagnostic biopsy	67 (36.2)	37 (41.1)	7 (20.0)	23 (38.3)	
MDT discussion, n (%)	452 (80.3)	227 (80.2)	60 (80.0)	165 (80.5)	0.995

SD = standard deviation; BMI = body mass index; a-CCS = age-adjusted Charlson Comorbidity Index Score; GR = growth rate; MDT= multidisciplinary team conference.

### Growth rate

**Figure 1:**
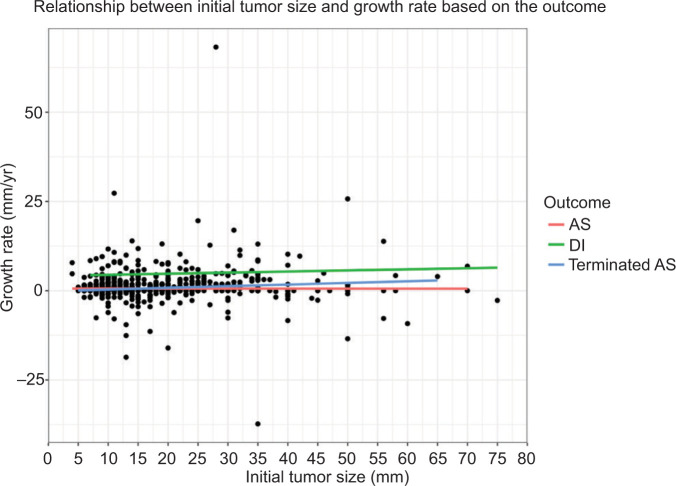
Growth rate as a function of initial tumour size stratified on the three groups. AS = active surveillance; DI = delayed intervention, Terminated AS = patients who terminated AS.

The overall mean GR was 1.3 mm/yr with significant variation between the groups: DI (4.8 mm/yr), AS (0.6 mm/yr) and terminated AS (0.9 mm/yr). We found a positive correlation between initial tumour size and GR (r=0.13, p=0.0011; Figure 1). Patients in the AS group demonstrated a consistent GR across all initial tumour sizes, whereas patients in the DI group and patients who terminated AS presented with higher GRs for larger initial tumour sizes. Compared to the other two groups, the DI group had higher GRs for every initial tumour size. The other groups demonstrated GRs close to zero for every initial tumour size (Figure 1).

### Initial follow-up

A total of 563 patients underwent their initial follow-up after a mean duration of 7.6 months. At the first follow-up visit, the average tumour size was 20 mm with a mean GR of 1.1 mm/yr from the time of deciding on AS to the first follow-up. Among the 563 patients, 43 patients (8%) underwent a biopsy during the first follow-up period, the main reason being tumour growth (58%). The decisions regarding DI versus continued AS were made by varying numbers of physicians, with variations observed both within and between centres. The AS programme of 125 patients (22%) was reassessed and discussed at MDT for further treatment consideration. Subsequently, a total of 31 patients (6%) transitioned to DI, 87 patients (16%) terminated their AS programme and 445 patients (79%) continued AS. Currently, 345 of the 445 AS patients have undergone their second follow-up (Table 2).

**Table 2:** Characteristics of the first five follow-ups.

	Follow-up 1 (n = 563)	Follow-up 2 (n = 345)	Follow-up 3 (n = 185)	Follow-up 4 (n = 101)	Follow-up 5 (n = 57)
Mean time between follow-ups (month) (SD)	7.6 (3.4)	9.9 (4.8)	11.6 (5.4)	11.5 (6.8)	10.4 (4.3)
Mean tumour size/mm (SD)	20 (12.6)	21 (13.4)	22 (17.9)	25 (14.2)	27.6 (15.5)
Mean GR since the last follow-up (mm/yr) (SD)	1.1 (10.6)	1.0 (7.6)	1.5 (7.7)	1.7 (5.6)	2.2 (4.5)
Biopsy, n (%)	43 (7.6)	19 (5.5)	13 (7.1)	7 (6.9)	7 (12.3)
Reason for biopsy, n (%)					
Tumour growth	25 (58.1)	12 (66.7)	9 (69.2)	6 (85.7)	5 (71.4)
Health improvement	10 (23.3)	–	1 (7.7)	–	–
Patient’s desire for treatment	3 (7.0)	–	–	–	–
Former failed biopsy	3 (7.0)	3 (16.7)	–	1 (14.3)	1 (14.3)
Changed morphology	2 (4.7)	3 (16.7)	3 (23.1)	–	1 (14.3)
MDT discussion, n (%)	125 (22.2)	67 (19.4)	43 (23.4)	21 (20.8)	11 (22.4)
Overall treatment decision, n (%)					
Delayed intervention	31 (5.5)	18 (5.2)	12 (6.5)	6 (5.9)	4 (8.2)
Terminated AS	87 (15.5)	57 (16.5)	23 (12.4)	19 (18.8)	8 (16.3)
Continued AS	445 (79.0)	270 (78.3)	150 (81.1)	76 (72.2)	37 (75.5)

SD = standard deviation; GR = growth rate; MDT = multidisciplinary team conference; AS = active surveillance.

**Figure 2:**
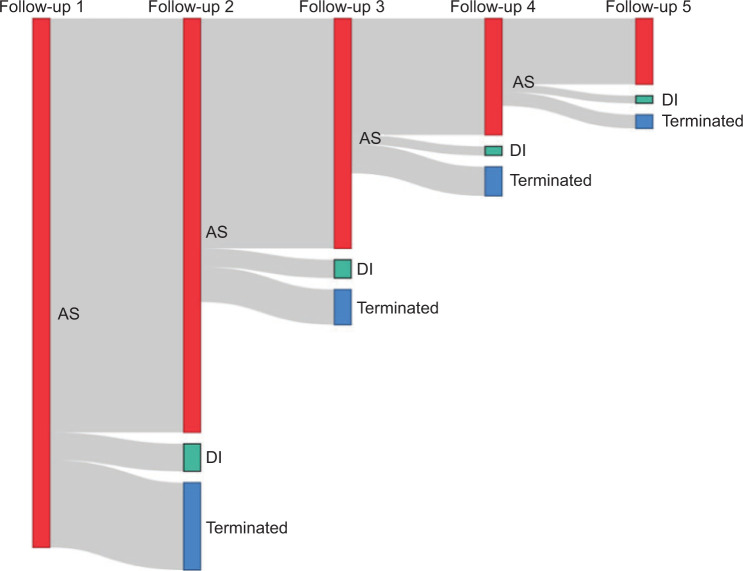
The flow of patients at each follow-up visit. AS = active surveillance; DI = delayed intervention; Terminated = patients who terminated AS.

During the initial five follow-ups, a progressive increase in tumour size was observed, with a positive correlation between tumour size and time (r=0.0705, p=0.00157). In addition, during the initial five follow-ups, approximately 7% of patients underwent a biopsy, primarily due to tumour growth (∼70%). However, approximately 60% of the biopsied patients continued their AS programme, mainly due to a high non-diagnostic biopsy rate (∼40%) and comorbidities (∼35%). Table 2 provides detailed characteristics of the initial five follow-ups. The cohort is different, with a decreased number of patients at each subsequent follow-up due to DI and AS termination (Figure 2).

### Delayed intervention

A total of 75 patients (13%) underwent DI after an average follow-up of 18 months. The mean initial tumour size was 20 mm and the mean GR was 4.8 mm/yr. The primary reasons for DI were a GR >5 mm/yr (59%) and biopsy-verified cancer during follow-up (24%). Other reasons included health improvement (8%), elective crossover (4%), changes in morphology (4%) and development of symptoms (1%). Cryoablation was the most frequently utilised treatment modality (65%), followed by partial nephrectomy (19%) and nephrectomy (16%).

Only one patient developed metastasis during AS. The patient initially presented with a 28 mm biopsy-verified clear cell renal cell carcinoma (ccRCC), Fuhrman grade 2. AS was recommended because the tumour did not qualify for cryoablation and was expected to present with slow growth. However, at the first follow-up after 6 months, the tumour had significantly grown to 64 mm, and imaging revealed suspicious findings of metastasis in both the biopsy canal and the lungs. Subsequently, the patient underwent an open nephrectomy, and the pathology report confirmed pT3a ccRCC, Fuhrman grade 3, with 60% sarcomatous and necrosis features, along with biopsy-confirmed lung metastasis.

### Terminated AS programme

A total of 205 patients (36%) terminated AS after a mean follow-up of 22 months. The initial mean tumour size was 20 mm and the mean GR was 0.9 mm/yr. The primary reasons for AS termination were comorbidities (33%) and a benign morphology (22%) during follow-up. Patients who terminated AS due to comorbidities presented primarily with the same CCI score at termination as at diagnosis. Other reasons included death due to non-kidney cancer causes (17%), slow or no growth (10%), patient preference (9%), advanced age (4%), follow-up at another department due to another cancer diagnosis (3%) and loss to follow-up (2%).

## Discussion

No standardised protocol is available with respect to an optimal follow-up schedule or imaging modalities for AS of SRMs, and both guidelines and studies offer considerable variations (16). This study was no exception, as the participating centres did not adhere to a standard protocol. Instead, the AS programme was individually customised for each patient, but no major variations were observed. The Delayed Intervention and Surveillance for Small Renal Masses (DISSRM) protocol was designed with imaging every 6 months for 2 years and then annually afterwards (9), which aligns with the guidelines of the American Urological Association and the European Association of Urology (17, 18). In contrast, the Canadian Urological Association suggests CT or MRI every 3 months in the first year, every 6 months in the next 2 years and annually thereafter (19). This approach is supported by some of the largest reported series to date (20). In the present study, the mean time to first follow-up was 7.6 months, exceeding the aforementioned guidelines. Subsequent follow-ups within the first 5 years had imaging intervals ranging from 9.9 to 11.8 months, which deviate from the guidelines’ recommended intervals for the first 2 years. However, we observed an overall low GR, which suggests longer time intervals after the initial phase and may presumably be the cause of longer follow-up intervals for the patients.

The role of initial renal tumour biopsy (RTB) in the context of AS remains controversial, with concerns regarding diagnostic accuracy, safety and its impact on clinical management. In our cohort, the initial RTB rate was 33%, which is slightly higher than the DISSRM protocol, which had an initial RTB rate of 20% in recent years (21). Our study noted a 36% inconclusive rate, surpassing the 10%–20% range reported in other studies (22), but is consistent with Lechevallier et al., who reported an inconclusive RTB rate of 37% for tumours <3 cm (23). Within our study, approximately 7% of all patients underwent a biopsy at each follow-up visit within the first 5 years. Remarkably, 50% of the biopsied patients continued AS despite the biopsy, mainly due to a high inconclusive rate (40%) and comorbidities (35%). Consequently, the impact of RTB, both initially and during follow-up, appears to be relatively modest. Therefore, it is important to consider which patients are offered RTB.

We found a mean overall GR of 1.1 mm/yr with a median follow-up of 21 months, which is consistent with a previous prospective study by Organ et al., who reported a GR of 1.2 mm/yr with a mean follow-up of 20 months (14). Other studies have reported higher GRs of 2–3 mm/yr (24). Growth kinetics are often considered to be the main factor for initiating active treatment, and multiple studies have demonstrated a correlation between GR and tumour aggressiveness, making it a valuable parameter for patient follow-up (16, 25). Consistent with these findings, our results revealed a GR of 4.8 mm/yr in the DI group compared to 0.6 mm/yr in the AS group. In addition, GR was the primary trigger for intervention in more than half of cases. However, some argue that GR alone may not be sufficient to predict malignancy, as benign masses can present similar GRs as malignant lesions (26).

The size of SRMs is widely acknowledged to serve as an indicator of their potential malignancy. Rothman et al. conducted a large cohort study and demonstrated a 13% increase in the likelihood of high-grade tumours for every 1 cm increase in initial tumour diameter (27). In line with these findings, the DI group in this study had a larger initial tumour size than the AS group. However, Kouba et al. (28) found no correlation between GR and initial tumour size, and our patients with terminated AS presented with the same initial tumour size as the patients with DI.

The incidence of DI in our study was 12%, with a median follow-up of 12 months. These findings are consistent with previous studies (10, 16). A wide range of progression rates to DI have been reported in the literature, varying from <5% to as high as 65% (29, 30). A pooled analysis by Gupta et al. (31) revealed that DI patients were significantly younger than those continuing with AS (64 vs. 72 years), which is consistent with our findings (69 vs. 72 years). Gupta et al. also reported a mean GR of 7.0 mm/yr for DI patients, surpassing the GR of 4.8 mm/yr observed in our DI group. The primary trigger for intervention in our cohort was a GR >5 mm/yr, which aligns with the findings reported by Rebez et al. (16).

A total of 205 patients (36%) in our study terminated AS after a median duration of 13 months. The termination was primarily attributed to comorbidities (34.8%), benign morphology during follow-up (21.5%), and death due to non-kidney cancer causes (16.6%). The notably high termination rate, especially due to comorbidities, which remained consistent throughout the AS period for most patients, and non-kidney cancer-related deaths, suggests that some patients may not have been ideal AS candidates initially. This trend points towards a deviation from AS principles, leaning more towards watchful waiting given the limited treatment options for these patients and in case of progression during follow-up. These findings support the need for an improved understanding of AS patient profiles and reveal a lack of clarity and precision in patient selection for AS. Nonetheless, all patients in this cohort adhered to a protocol that included scheduled imaging. In line with the existing literature, which reports an overall risk of metastasis of 1% to 2% with intermediate-term follow-up (32), only one patient developed metastasis during AS.

Our study has some limitations that should be acknowledged. The observational design of the study introduces the possibility of confounding factors and selection bias. The selection bias was further compounded by the challenge of identifying AS patients, as there is no specific diagnosis code for the AS procedure. The study included patients from three Danish centres, but the distribution of patients across these centres was uneven, potentially influencing the data. The study primarily included elderly patients with SRMs, which limits the generalisability of the findings about younger patients and those with larger masses. Furthermore, the majority of patients had a high comorbidity burden, likely rendering the AS programme and renal mass irrelevant to their overall prognosis. Finally, the study’s follow-up was relatively short, limiting the assessment of long-term metastasis and delayed intervention rates. Despite these limitations, the present study provides valuable insights into a large group of patients and offers details on patients in a current AS programme, those who underwent DI, and those who terminated AS.

## Conclusions

Active surveillance appears to be a safe initial management strategy for SRMs. This conclusion is supported by an overall low GR and the occurrence of metastasis in only one patient. Patients undergoing DI were younger and presented with a larger initial tumour size and higher GR than those who continued with AS. A considerable number of patients terminated AS due to non-kidney cancer causes, highlighting the possibility that certain individuals enrolled in AS may not initially have been ideal candidates. The patient selection for AS appears inconsistent, emphasising the need for improved criteria to identify appropriate candidates, particularly considering comorbidities and the possibility of subsequent active treatment in the event of progression.
